# Unlocking STEM Identities Through Family Conversations About Topics in and Beyond STEM: The Contributions of Family Communication Patterns

**DOI:** 10.3390/bs15020106

**Published:** 2025-01-21

**Authors:** Remy Dou, Nicole Villa, Heidi Cian, Susan Sunbury, Philip M. Sadler, Gerhard Sonnert

**Affiliations:** 1 STEM Transformation Institute, Florida International University, Miami, FL 33199, USA; nvilla@fiu.edu; 2 Maine Mathematics and Science Alliance, Augusta, ME 04330, USA; hcian@mmsa.org; 3 Department of Science Education, Harvard-Smithsonian Center for Astrophysics, Cambridge, MA 02138, USA; ssunbury@cfa.harvard.edu (S.S.); psadler@cfa.harvard.edu (P.M.S.); gsonnert@cfa.harvard.edu (G.S.)

**Keywords:** STEM identity, family, caregivers, conversations

## Abstract

Research shows that family conversations about STEM topics positively influence children’s STEM identity development. This study expands on these findings by exploring how family conversations *beyond* STEM content contribute to this development. Specifically, we focus on how non-academic forms of family support—as described by students who face systemic racial discrimination in STEM—shape these conversations. In this way, we extend existing work by exploring the extent to which families’ dispositions to talk about a wide range of topics—not just in STEM—might further support youth identification with STEM fields. Using Family Communication Patterns Theory (FCPT) to guide our analysis, we examined data from a survey of first-year college students (*n* = 1134) attending Minority-Serving Institutions and public universities in the United States. The survey asked students to reflect on their childhood conversations and their current sense of identity in STEM. Using structural equation modeling, we found that family disposition to engage in conversations about a broad range of topics was linked to more frequent STEM-related conversations during childhood and, in turn, greater identification as a “STEM person” in college. These findings highlight the complex ways that family communication patterns can support construction of an individual’s sense of themselves as a STEM person in later years. By interpreting these findings using FCPT, we highlight the nature of family communication patterns that can contribute to STEM identity formation.

## 1. Introduction

Researchers have emphasized the importance of attending to learners’ holistic selves when considering children’s developing affinities toward STEM and STEM-related career aspirations (Buschor et al., 2014; Carlone & Johnson, 2007; Collins, 2018; Paul et al., 2020; Rahm & Moore, 2016). “STEM identity”, in particular, has been embraced as a useful construct for understanding and addressing barriers to youth who have been traditionally marginalized from STEM participation by racially exclusive notions of what “kind of person” does STEM—notions that in effect exclude Black and Latino learners from authentically engaging in the STEM enterprise (Barton & Tan, 2010; Master, 2021; McGee, 2016). A growing line of evidence points toward family contexts as spaces where children’s budding dispositions toward STEM are likely to be nurtured in greater harmony with their evolving social identities (Belgrave et al., 2022; Castañeda et al., 2022; Pattison et al., 2022). Much of this research frames caregivers—across cultural backgrounds—as agentic actors in the development of children’s STEM identities, regardless of their formally recognized STEM-related expertise (Cian et al., 2022; Ennes et al., 2023). [Unless specifically clarified, here and throughout the text, we refer to “STEM” as a loosely defined set of domains, disciplines, and vocations that are discursively associated with the terms “science”, “technology”, “engineering”, and/or “mathematics” (e.g., Shein & Tsai, 2015). In this way, we primarily reflect how the term is used in reports by federal agencies (e.g., (Committee on Science, Technology, Engineering, and Mathematics, 2024)), by academic organizations (e.g., (National Academies of Sciences, Engineering, and Medicine, 2022)), and in formal or informal schooling contexts (e.g., National Academy of Engineering & National Research Council, 2014). When alluding to a particular study, we use the terminology relevant to that study (e.g., “science”, “physics”, “STEM”)]. 

Studies of how children’s socialization occurs in family contexts (i.e., how children’s self-knowledge develops) point toward their imitation of caregivers’ behaviors and conversation patterns (Gergely & Watson, 1999) as potential grounds for examining STEM identity development. While socialization can take on many forms, Dou et al. (2019) found that having frequent childhood conversations about science was more closely associated with college students’ (*n* = 15,847) identification as a “STEM person” than participation in out-of-school STEM learning experiences, even when controlling for parental education level, childhood interest in STEM, gender, and self-reported racial categorizations. Similar research (Marotto & Milner-Bolotin, 2018; Stewart, 2022) confirms this, indicating that family communication surrounding STEM topics may facilitate identity development in ways that formal and non-formal learning experiences may not.

Consequentially, imperatives that foreground STEM participation and related career pursuits could benefit from cross-disciplinary theories that encapsulate both family socialization and STEM identity development. Two important understandings offer grounds for this: (1) there is a strong relationship between individuals’ identification with STEM and their pursuit of STEM-related occupations and (2) evidence points to children’s familial socialization as foundational to their STEM identity development. However, models that encapsulate the socialization process are scarce. Given the societal goals that at times accompany these imperatives (Committee on Science, Technology, Engineering, and Mathematics, 2024), models must also account for varied cultural and familial contexts, and attention must be afforded to the experiences of individuals systemically marginalized in STEM fields. This is particularly true for Black and Latino youth who encounter discriminatory barriers that significantly challenge their pursuits (Barton & Tan, 2010; Master, 2021; McGee, 2016) in light of evidence indicating that those who persist often credit conversations with caregivers as fueling that persistence (Cian et al., 2022; McGee & Bentley, 2017; Yosso & Burciaga, 2016).

To test a model of STEM identity development that attends to familial socialization and support among youth, we examined how the frequencies of family conversations about and beyond STEM topics during childhood were related to college students’ identification with STEM. Specifically, we were guided by the following research question: to what extent does the frequency of childhood conversations with caregivers about STEM topics mediate the relationship between the frequency of conversations about broader topics and STEM identity measures of college students attending Minority-Serving Institutions (MSIs) while accounting for shared family interest in STEM? (See Section 4.1 for a conceptual illustration). We employed Family Communication Patterns Theory to guide our model’s development due to its relevance for our research aims, as well as it having shown the ability to exhibit explanatory power across culturally diverse populations (Koerner & Schrodt, 2014). We recruited students attending federally designated Historically Black Colleges and Universities (HBCUs) and Hispanic-Serving Institutions (HSIs) to center our work in the childhood experiences of Black and Latino youth (McGee, 2016).

## 2. Literature Review

### 2.1. Marginalized STEM Identities

STEM identity is often heuristically described as a measure of an individual’s self-perception as a “STEM person” (Dou & Cian, 2022; Simpson & Bouhafa, 2020). However, much nuance has been afforded in the conceptualization of this construct, being generally understood as multifaceted, encompassing how individuals consciously and unconsciously position themselves and others within communities associated with STEM (Kim et al., 2018; Simpson & Bouhafa, 2020; Vedder-Weiss, 2018). As such, its development is often situated or implicitly embedded (e.g., Carlone & Johnson, 2007; Holland et al., 1998) within the context of Social Identity Theory (Tajfel, 1978), attending to the relational interactions that shape identification with STEM. This relational component is reflected in research suggesting that children’s understanding of who constitutes a STEM person is often informed by significant adults, such as caregivers and educators, as well as informal learning experiences, including television and online media consumption, afterschool programming, and institutions such as zoos and science centers (Dou et al., 2019; Dabney et al., 2013; Maltese & Tai, 2010; Shenouda et al., 2024; Steinke, 2017).

The socialization that children experience in formal and informal learning contexts explains the durability of taken-for-granted archetypes of a “STEM person” (Cian & Dou, 2024; Garriott et al., 2017; Verdín et al., 2018). Common archetypes of STEM professionals adhere to narrow sets of characteristics that are predominantly embodied by White, masculine identities (Kayumova & Dou, 2022; Mensah & Jackson, 2018; Rosebery et al., 2010), appearing in portrayals of STEM that children often encounter in media (Steinke, 2017) and other social or parasocial contexts. The plethora of “Draw-A-Scientist” studies reflect children’s assimilation of these archetypes across a variety of age groups (Ferreira & Valente, 2024; Finson, 2002) and countries of origin (Ferguson & Lezotte, 2020). As a result, children must contend with their embodiment of the characteristics of these archetypes when considering their identification with STEM (Master, 2021).

For children who do not identify with these archetypes, the misalignment between their social identities and the social expectations that they encounter in STEM contexts can pressure them to suppress aspects of their identities in order to belong or drop their STEM pursuits altogether (McGee, 2016). In response to national calls for increasing racial and gender diversity in the workforce as a means of remaining competitive in a global economy (Committee on Science, Technology, Engineering, and Mathematics, 2024), researchers point to these identity-related conflicts that racially marginalized youth encounter (Flores et al., 2024; Ortiz et al., 2019) as reasons for the racial homogeneity found in the national STEM workforce (National Science Board, 2024). Even when youth from non-dominant communities come to identify with STEM through the pursuit of a college degree in a STEM field, they face “racially hostile academic spaces” (McGee, 2016, p. 1626), requiring them to contend with stereotyping and microaggressions that oppress their linguistic and cultural identities. For instance, Smith et al. (2023) note this occurring among Latine engineering undergraduates, as students recalled being ignored or directly discouraged by their professors, found themselves to be the butt of jokes (e.g., “you’re in construction engineering because you’re Mexican”, p. 2357), and heard suggestions that their participation in engineering programs was a result of race- or ethnicity-related scholarships rather than merit.

### 2.2. Conversations About STEM Topics

Family STEM-related interactions evidence the cognitive benefits of conversations with caregivers that occur outside formal learning experiences. Caregivers’ explanatory talk (i.e., responses to “Why” questions) and elaborative talk (i.e., addressing open-ended “Wh-” and “How” questions) in science museums have been shown to support children’s scientific reasoning (Booth et al., 2020; Callanan & Jipson, 2001), recall of STEM content (Haden et al., 2014), and exploratory behavior (Callanan et al., 2020). Related findings have been observed in other, out-of-school time contexts (Fender & Crowley, 2007). For example, Leichtman and colleagues (Leichtman et al., 2017) found that children who discussed school science lessons at home with parents recalled more of the content several days later, and their ability to recall information was shown to be partially mediated by the characteristics of their conversation even when parents were naïve to the content.

In addition to these cognitive outcomes, research studies document how parent–child interactions facilitate STEM identity development (e.g., Allen & Weidler-Lewis, 2023; Cian et al., 2022; Hernandez et al., 2016; Pattison et al., 2020; Šimunović & Babarović, 2020) in ways that are consistent with dialectical models of socialization (Kuczynski & De Mol, 2015):

*Parents initiate trajectories by selecting environments that expose children to experiences and invest resources in particular activities, such as music lessons that may or may not pan out. Parents may support trajectories through proactive and sustained efforts, including encouragement, time, and helpful messages and material assistance. Parents mediate trajectories that are chosen by the child by helping the child to interpret roadblocks and helping them to avoid problematic trajectories. Finally, parents provide guidance by reacting positively or negatively to child-initiated trajectories by supporting the child’s choices of activities, educational and careers[,] or using their power to attempt to redirect or create barriers to the child’s choices*.

(p. 55)

Evidence from retrospective studies of college students support the overlap between dialectical socialization in families and STEM identity development, finding that those who reported higher frequencies of childhood science talk with caregivers or close family members were more likely to self-identify as a “STEM person” and aspire to careers in STEM fields (Dou et al., 2019; Dou & Cian, 2021). Furthermore, Jackson et al. (2019) highlight the particular value of parent–child conversations for affirming women’s science and science career interests through social recognition. Similarly, when exploring family encouragement of scientific pursuits, Stake (2006) reported a positive association between high school students’ self-reports of family encouragement and their attitudes toward science, their science confidence, and their perception of themselves as a “successful future scientist” (p. 1037).

### 2.3. Caregiver Conversations Beyond STEM

The resources that are assumed to support youths’ STEM identity development (i.e., “STEM capital”) are often narrowly defined and exclusionary, such as connections to professionals in science-related roles and visiting informal STEM learning institutions like museums and zoos (Moote et al., 2020), which can be cost-prohibitive (Dawson, 2014). These perspectives presume that STEM identity is supported when families can offer access to resources directly related to STEM and STEM careers. However, much of the family support described in studies carried out with Black and Latino college students suggest that the characteristics of family interactions that influence their choices to enroll in and complete undergraduate STEM programs extend, at least in part, beyond these kinds of experiences, both in terms of context and content. For example, Ortiz et al. (2019) document the importance of family encouragement for Black college STEM majors, characterizing it as “emotional labor” (p. 317) rendered by caregivers and close kin that buoys students’ emotional success. Similarly, Frederick et al.’ (2024) longitudinal work with first-generation Latino students highlights the link between family encouragement and STEM career persistence, particularly during transition periods (i.e., during summer bridge programs and post-degree obtainment). Interviewees in their study similarly described benefits gained from this kind of encouragement even when “their parents were unable to offer them concrete advice about their education or career plans” (p. 7).

While the nature of these interactions with family members may be tangentially related to traditionally understood notions of STEM capital, they offer concrete benefits to STEM students by “instilling emotional perseverance, providing reassurance, and being on-going sources of inspiration” (McGee & Spencer, 2015, p. 485). Frederick et al. (2024) discuss how these familial interactions may include encouragement, advice, and expressions of collective pride that anchor students in their “unique cultural values” (p. 6). The value of these interactions to attenuate the socioemotional and psychological challenges that Black and Latino students face is of such benefit that students oftentimes seek to recreate these forms of support by developing academic relationships that reflect their “familial cultural values of collectivism and familismo” (Frederick et al., 2024, p. 149). In this way, views of family conversations as contributing to STEM identity solely through the consideration of STEM-specific conversations may exclude other important ways that families engage with youth to support their identification with STEM, particularly among marginalized youth.

## 3. Theoretical Frameworks

In order to form an understanding of the extent to which family conversations about and beyond STEM shape college students’ STEM identities, we ground ourselves in two theoretical frameworks: STEM identity development theory and Family Communication Patterns Theory. These frameworks are linked by their dependence on social interactions often (though not always) carried out in conversational contexts. While we center STEM identity development theory in our conceptual approaches, we draw on Family Communication Patterns Theory in our research design, model development, and interpretation of results.

### 3.1. STEM Identity Development

Our work is grounded in a social theory of STEM identity development (Kim et al., 2018) that identifies STEM interests, recognition from others, and perceptions of performance–competence as primary elements of STEM identity (Carlone & Johnson, 2007; Cian et al., 2022; Hazari et al., 2010). According to this framework, an individual’s self-identification with STEM domains (e.g., “science”), STEM disciplines (e.g., “physics”), or STEM as a whole grows through the cultivation of interests, acknowledgment from others, and a sense of their own ability to perform related tasks (Cian et al., 2022). This perspective emphasizes the social nature of identity formation, whereby self-identification continuously evolves through affirmations of interest (Jackson et al., 2019), as well as recognition from field authorities, such as teachers or scientists, and caregivers (Kim et al., 2018). This framework conceptualizes identity as a dynamic, language-based construct that allows individuals to author and adapt their identities in response to situational factors (Gee, 2000; Gee et al., 2001). Riedinger (2015) illustrates the contextual nature of STEM identity development in her descriptions of Brynn and Hannah, two middle school students attending a 4-day residential science camp. These students juxtaposed their science classroom performances with those of Dale—a classmate they recognized as a “very good [science] student” (p. 464)—describing Dale as an “overachiever” who tended to have all the answers to their teacher’s questions and positioning themselves as “average” by comparison. However, as Brynn and Hannah were presented with opportunities to explore their interests outside of the formal setting of their science classroom, the authors describe how the two girls began to use more assertive language that positioned them in closer alignment with “good science learners” (p. 463).

### 3.2. Family Communication Patterns Theory

Considered a grand theory of family communication, Family Communication Patterns Theory (FCPT) offers a model for understanding how members of a kinship group come to develop “shared social realities” (Koerner & Schrodt, 2014, p. 2) through interactions. While FCPT attends to its namesake—the patterns that govern communication between family members—the primary aim of its development lies in “explaining how parents socialize their children to process information stemming from outside the family” (Koerner & Fitzpatrick, 2006, p. 51). Its application in empirical studies has proven fruitful over the years (Schrodt et al., 2008), exhibiting an average correlation of r = 0.29 (SD = 0.18; *n* = 56 studies), with outcome variables classified into the following categories: information processing (e.g., cognitive flexibility, political identity, skepticism), behavioral (e.g., shopping patterns, parental affection, family conflict), and psychosocial (e.g., self-concept, mental health symptoms, anxiety). In part due to its development in the field of communication science, relatively few studies have applied FCPT in service to STEM education research, albeit with some exceptions (e.g., scientific literacy; Pingree et al., 2000). However, its utility in explaining youth identity development along similar dimensions (e.g., Bosch et al., 2012; Horstman et al., 2016; Phillips et al., 2018) foreground our application of FCTP to understanding STEM identity development.

FCPT posits that family communication patterns about objects in their physical and/or social environment shapes members’ beliefs and attitudes about those objects (Koerner & Schrodt, 2014). In families, this process—referred to as “co-orientation”—tends toward agreement through two primary dispositions: “conversation orientation” and “conformity orientation” (Schrodt & Scruggs, 2021). Conversation orientation represents the degree to which members of a family engage in conversations about a broad range of topics in high frequency with minimal constraints (Koerner & Fitzpatrick, 2006). Relatedly, conformity orientation represents the degree to which family communication practices emphasize “homogeneity of attitudes, values…beliefs, harmony, conflict avoidance, and the interdependence of family members” (p. 55). FCPT suggests that the intersection of where families lie along these two dimensions partially explains the extent to which family members develop shared beliefs and attitudes (i.e., co-orientation).

According to Koerner and Fitzpatrick (2006), children’s tendencies to adopt their parents’ belief systems result from patterns in their family’s conversation and conformity orientations (see Table 1). Parents in families with high conversation orientation, as well as high conformity orientation, value their children’s consensual agreement with their beliefs or decisions, which they foster through open communication, conflict resolution, and willingness to explore children’s ideas. Members of families with high conversation orientations but low conformity orientations (i.e., pluralistic families) seek one another’s opinions about “a wide range of topics” (p. 7) without pressure to agree with one another’s beliefs or decisions, which may differ from one family member to another. On the other hand, families with low conversation orientations and high conformity orientations tend to place great emphasis on agreement, offering little room or concern for ideas that differ from those of the head(s) of household, while families with both low conversation and low conformity orientations tend to show little interest in one another’s decisions and opinions.

## 4. Materials and Methods

### 4.1. Research Motivations

**Figure 1:**
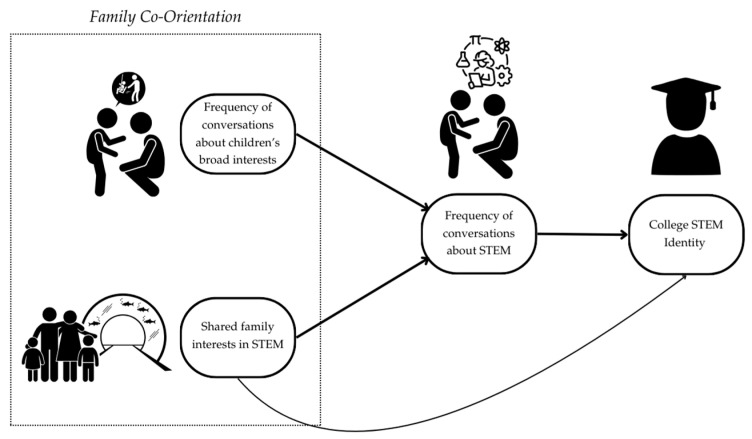
The illustration above represents our conceptualization of how Family Communication Patterns Theory (FCPT) overlaps with youth STEM identity development theory. STEM identity theory posits children’s socialization as a major factor in its development. This socialization is catalyzed by caregiver–child conversations and engagement in shared experiences. The FCPT concept of co-orientation offers a framework for parsing cultural differences across families that result in varying conversation and conformity orientations that can, in turn, shape children’s beliefs about STEM and about themselves. For simplicity, we use one-sided arrows; however, much research suggests reciprocal relationships across the factors represented in the illustration.

Our research was motivated by prior multi-method work with college students pursuing STEM majors at a federally designated Hispanic-Serving Institution (HSI) in the southeastern region of the U.S. Inspired by findings revealing significant associations between the frequency of childhood conversations with caregivers about STEM and measures of students’ STEM identity (Dou & Cian, 2021), we carried out follow-up interviews with respondents to understand the content, context, and structure of those conversations (Cian et al., 2022). Across these interviews, we noted conceptual overlaps between co-orientation as outlined in FCPT (Koerner & Fitzpatrick, 2006) and youth STEM identity development. For example, caregivers’ willingness to engage in conversations about topics that interested their children, regardless of the topics’ association with STEM, resonated with the communication patterns of families with high conversation orientations. We also noted a general tendency for college students to describe post-secondary degree obtainment as an unquestioned pursuit that suggested high conformity orientation with parental values, even when parents expressed differences of opinion as to the value of specific degree programs (e.g., Education major). These overlaps prompted us to consider the utility of combining the two frameworks (see Figure 1).

The study presented below represents an initial effort to empirically explore conceptual overlaps between FCPT and STEM identity theory. Specifically, we sought to understand the extent to which quantitative relationships exist between family conversation orientation, engagement in conversations about STEM, and STEM identity. We tested a structural equation model of these measures to examine hypothesized relationships between them. In doing so, we sought to encompass family communication patterns that may shape youth STEM identity through the development of shared beliefs and attitudes toward STEM.

### 4.2. Sampling

Data were collected at a single time point through a survey administered to students (*n* = 1134) primarily attending HBCUs, HSIs, and/or large public universities in the United States (U.S.). The development of the survey originated in a broader study funded by the National Science Foundation (Award No. 2215050) to explore the out-of-school learning experiences of youth who identify with under-represented groups in STEM fields. In order to ensure the representation of students across a spectrum of career interests and pursuits (i.e., both STEM and non-STEM majors), survey respondents were recruited through first-year required courses at their respective institutions. The survey invited students to respond to items measuring a variety of variables, including career aspirations, prior schooling performance (e.g., the number of advanced mathematics and science courses taken, high school grade point average), participation in out-of-school learning activities (e.g., attending camps, visiting museums, playing with at-home chemistry kits), dispositions toward STEM (e.g., interest in STEM topics, self-perception as a STEM person), and family experiences related to STEM (e.g., caregiver involvement in STEM professions, family support of after-school STEM activities). For the current study, a subset of these items was selected for analysis. See Table 2 for a descriptive summary of the sampled population.

### 4.3. Measures

#### 4.3.1. STEM Identity

We measured STEM identity using a 9-item scale developed by Hazari et al. (2010) and later modified and validated in the context of “STEM” (Dou & Cian, 2022). The items aimed to measure three primary components of STEM identity: interest, recognition from others, and performance–competence. Respondents were invited to rate their level of agreement with statements attending to these components (e.g., interest: “I enjoy learning STEM”; recognition: “My family sees me as a STEM person”; performance–competence: “I feel confident in my ability to learn STEM”) using a 6-point Likert scale (ranging from 0 to 5) anchored only at the poles (i.e., Strongly Disagree to Strongly Agree). Item means ranged from 1.98 to 2.50 (SD = 1.70–1.92) with a scale midpoint of 2.50. As in previous studies (e.g., Dou et al., 2019), we offered no formal definition of the term “STEM” other than a list of domains represented by the acronym (i.e., science, technology, engineering, mathematics) on the first page of the survey. We chose this approach to ground the interpretation of the term in respondents’ self-constructed understanding. Confirmatory factor analysis supported the theorized three-factor structure of the items (SRMR = 0.02, TLI = 0.98, RMSEA = 0.08, BIC = 20,303) with all item factor loadings at or above 0.90. Tests of internal reliability resulted in calculations of Cronbach’s alpha ranging from 0.94 to 0.96 (CI_95%_ = ±0.01) across the three factors. Respondent STEM identity (*M* = 2.23, SD = 1.59) was specified as a latent variable indicated by the means of the items associated with each of the components (i.e., interest, recognition from others, and performance–competence).

#### 4.3.2. Conversational Frequencies

We conceptualized conversation orientation as the frequency of engaging in conversations about broad sets of topics (i.e., Broad Topics Talk) with caregivers during childhood. We drew on prior survey items devised to measure conversation orientation (Schrodt & Scruggs, 2021) and language used by participants in prior studies to develop a single-item variable. Our use of a single item was predicated on measuring conversation orientation as an observed variable (i.e., recollection of prior experience) as opposed to a latent variable. Specifically, respondents were instructed to indicate how often they participated in the following activity: “Talk about any topic I found interesting with my parent(s)/caregiver(s)”. Using a Likert scale anchored at the poles (“Not at All” to “Very Often”), responses ranged from 0 to 5 with a mean of 2.89 (SD = 1.73) and a median of 3.

The frequency of conversations with caregivers about STEM topics (i.e., STEM talk) was similarly measured as an observed variable using a single, 6-point Likert scale item (i.e., 0 to 5) anchored at the poles. Content and criterion validity of this item was established in prior studies referenced above. Using the statement “Talk about STEM topics with my parent(s)/caregiver(s)”, responses ranged from 0 (“Not at All”) to 5 (“Very Often”) with a calculated mean of 1.72 (SD = 1.72) and a median of 1. The STEM talk item was presented to respondents after the Broad Topics Talk item.

#### 4.3.3. Family Interest in STEM

Respondents were asked to report their perceptions of their family’s interest in STEM by indicating whether or not “STEM is a family interest”. This item was included in a set of similar items that respondents were invited to mark if the item described their perception of their “family’s interest in, and attitudes toward, STEM”. Responses to “STEM is a family interest” generated a binary output of 0 or 1 (i.e., unmarked and marked, respectively), with the former indicating no perception that STEM is a family interest and the latter indicating agreement with the statement. In order to avoid miscoding missing responses as non-responses, an additional item was included that invited respondents to indicate that “None of these apply to me”.

### 4.4. Analysis

We selected structural equation modeling as our analytical approach given its utility for examining multiple directional relationships among variables and covariates simultaneously, as well as its robustness for handling partially missing data (Harlow, 2014). We specified a structural equation model that explored a mediated relationship between our three primary variables of interest (Broad Topics Talk, STEM talk, and STEM identity; see Section 4.3, “Measures”). We controlled for the effect of family interest in STEM, which we interpreted as a proxy for family disposition to engage in conversations about STEM topics. Our measure of STEM identity was treated as a latent variable indicated by three observed variables, i.e., STEM interest, recognition, and performance–competence (see Dou & Cian, 2022). Measures of conversational frequencies and perceived family interest in STEM were treated as observed variables. We estimated parameters using maximum likelihood estimation and tested the model using the lavaan package (Rosseel, 2012) in R (R Core Team, 2024). While qualitative and descriptive analyses of our variables did not indicate violations of statistical assumptions and we identified overall missingness below our 10% threshold (Bennett, 2001; Dong & Peng, 2013), we tested our model using a bootstrap of 10,000 draws to generate more robust confidence intervals for parameter estimates after carrying out single imputation (Rubin, 1987). The results from our analysis of imputed data did not meaningfully differ from those of non-imputed data.

### 4.5. Study Limitations

We framed our study as an initial effort in the exploration of STEM identity development in the context of family communication patterns given the conceptual overlaps between theories on these topics, corroborating research evidence, and potential for advancing knowledge through cross-disciplinary research. However, we acknowledge that this exploration is limited in the extent to which we directly attend to respondents’ conformity orientations. As such, we view findings from this study as potential prompts for the further integration of FCPT and STEM identity theory through research, rather than a comprehensive model of their integration.

Further, we recognize that despite our attention to respondents’ childhood experiences in relation to their present-time identification with STEM, the design of our study remains an associative one rather than a causal one. To the best of our ability, we avoided using language that implied causality (e.g., impact, determine) in favor of terms that highlighted associative relationships (e.g., correlate, shape). Causality, however, may have been inadvertently suggested by the normative conventions used when diagraming structural equation models (e.g., single-sided arrows).

## 5. Results

The test of our structural model achieved overall good fit with our data using thresholds described by Hooper et al. (2008): RMSEA ≤ 0.06; SRMR ≤ 0.06; TLI ≥ 0.96; CFI ≥ 0.96 (i.e., RMSEA = 0.01, SRMR = 0.01, TLI = 0.99, CFI = 0.99, BIC = 8703.62). Factor loadings for STEM identity indicators also indicated good fit with statistically significant (*p* < 0.001) values above 0.85 (Beauducel & Wittmann, 2005). We found no direct relationship between Broad Topics Talk and STEM identity. However, all remaining path estimates, including indirect effects, exhibited statistical significance at the *p* < 0.001 level.

**Figure 2:**
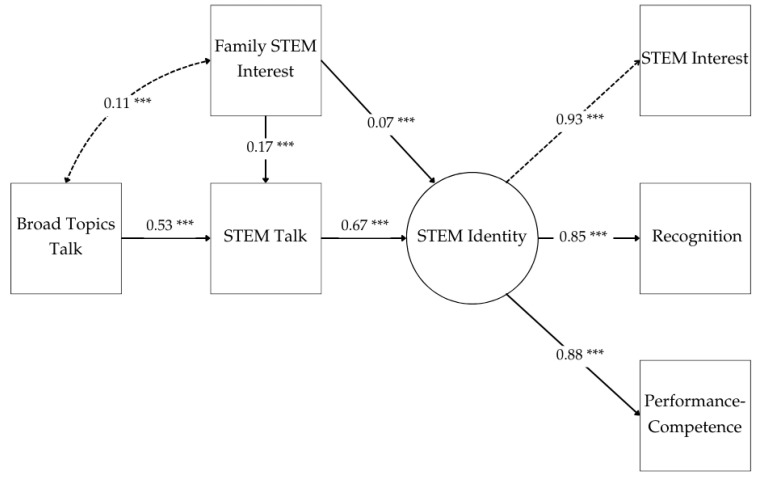
The path structure and standardized estimates of our structural equation model (RMSEA = 0.01, SRMR = 0.01, TLI = 0.99, CFI = 0.99, BIC = 8703.62). All path estimates were significant at the *p* < 0.001 level. Broad Topics Talk was not directly associated with STEM identity. *** *p* < 0.001.

We noted a high effect size in the relationship between STEM talk and measures of STEM identity (β = 0.67), confirming prior studies. We also noted a high effect size in the association between reported frequency of childhood conversations about “any topic” (Broad Topics Talk) and STEM talk (β = 0.53). This suggested a greater likelihood for engagement in STEM talk for respondents who more frequently engaged in Broad Topics Talk. In turn, Broad Topics Talk had a moderately sized indirect effect on STEM identity (β = 0.36) when mediated by STEM talk, such that a one-unit increase in Broad Topics Talk resulted in a 0.33 unit increase in STEM identity. In other words, respondents who indicated high conversation orientation were more likely report higher STEM identity as a factor of engagement in STEM talk. This was the case regardless of respondents’ perceptions of their family’s interest in STEM. However, respondents who also reported STEM as a family interest had a slightly higher likelihood of reporting high measures of STEM identity than those who engaged in STEM talk but did not indicate STEM as a family interest. We present all significant path estimates in Figure 2.

## 6. Discussion

Motivated by conceptual overlaps between family communication patterns and STEM identity theory, we explored college students’ STEM identity development through the lens of FCPT. Grounding our exploration in the experiences of individuals who identify with communities often marginalized in STEM contexts as a factor of race, we collected survey data from students enrolled at MSIs and/or public universities. Our findings revealed that students who reported frequent conversational engagement with caregivers about “any topic” (i.e., conversation orientation) during childhood were more likely to engage in conversations about STEM topics and, in turn, more likely to report high measures of STEM identity. This relationship exhibited a nearly three-to-one ratio whereby a three unit increase in Broad Topics Talk generally resulted in a one unit increase in STEM identity within a population of students pursuing both STEM and non-STEM degrees.

Because our model accounted for respondents’ perceptions of their family’s general interest in STEM and our sampled population included both STEM and non-STEM majors, our results offer quantitative evidence of mechanisms that can foster the development of children’s STEM identities even when youth’s interest in STEM differs from that of their caregivers. This finding stands in tension with research that frames the determinants of STEM identity exclusively within the confines of access to STEM-specific experiences and capital (e.g., Archer et al., 2012; Jones et al., 2021; Moote et al., 2020). Indeed, our measure of family STEM interest had the smallest effect on STEM identity (β = 0.07) compared to any other significant variable in our model, suggesting that this factor may be neither necessary nor sufficient for children to develop STEM identities. This is further supported by evidence de-emphasizing the need for caregivers to have STEM knowledge or sustained interest in STEM for nurturing their children’s STEM identities (e.g., Dotterer, 2022; Šimunović & Babarović, 2020; Solomon, 2003; McGee & Bentley, 2017) and research accentuating the emotional support and other non-academic familial resources that undergraduate STEM students draw on to sustain their STEM-related goals (e.g., Craig et al., 2018; Frederick et al., 2024; Ortiz et al., 2019).

Our findings have significant implications for the design of initiatives that aim to foster children’s STEM identities and related career pursuits. While many of these initiatives focus predominantly on children’s participation in STEM activities, they miss out on opportunities to involve caregivers and the impact these activities can have when they become topics of family conversations (Milner-Bolotin & Marotto, 2018). While researchers sometimes allude to parents’ lack of confidence to engage in conversations about STEM (e.g., Ansberry & Morgan, 2019), our findings provide new avenues for exploring family communication as a means to foster children’s STEM identity by leveraging parents’ dispositions to engage in conversations about children’s broad interests and experiences. For example, when recording the conversations of families participating in a self-guided nature walk, McClain and Zimmerman (2014) documented that families drew on their repertoire of shared experiences in the majority of their conversations and many of these conversations did not directly relate to STEM; in this way, when families discussed topics relevant to STEM content, such as identifying plant species, they also contextualized the content within their broader shared system of values and experiences. Reciprocally, other studies highlight the spontaneous ways that conversations about STEM can occur as a result of everyday family activities (e.g., Belgrave et al., 2022; Castañeda et al., 2022; Shirefley et al., 2020).

Family engagement programs (or program components) in formal or informal settings stand to benefit from attending to family communication practices. Family practices that foster open conversations between caregivers and children, regardless of the topic, hold the potential for children to explore their STEM-related interests, have those interests affirmed by significant authorities guiding their development, and be supported both instrumentally (e.g., enrolling a child in an after-school STEM program) and socio-emotionally (Cian et al., 2022). Whiston and Keller’s (2004) review of the literature summarizes a variety of ways that caregivers communicate support of their children’s interests and goals, including “showing interest and giving information, advice, suggestions, and feedback” (Whiston & Keller, 2004, p. 524). As such, there is a plausible likelihood that some of the goals of youth STEM programming can be achieved through family involvement that does not require parents to take part in or contribute directly to STEM activities by fostering open communication between parents and their children.

Taking a direct approach to fostering these kinds of conversation, Morris et al. (2023) found that by including simple “wh-question prompts” in popcorn-making instructions (e.g., “*Wh*at do you smell while the popcorn is popping?”, “*Wh*y did some of the kernels not pop?”), families engaged in 3 to 5 times more STEM talk than those who were encouraged to ask questions but did not receive prompts. While this study did not directly attend to identity development, the authors’ success in fostering family conversations without requiring parents to have prior STEM knowledge or providing scientific explanations had the effect of generating the kind of STEM talk that could potentially contribute to the development of their children’s STEM identities. Conversational prompts that attend to families’ everyday experiences offer valuable avenues by which to support family engagement “without requiring significant burdens related to cost and time” (p. 7) or highly incentivized changes to existing family practices.

Family communication pattern theory provides a meaningful lens through which to interpret our findings. Young et al. (2001) identified the nurturing of parent–child conversations—in their study about career goals—as strongly shaping their children’s dispositions toward certain careers. They also noted that these conversations were only effective when they relied on open communication, when caregivers aimed to align their goals with those of their children, and regardless of the participating families’ cultural backgrounds. The associative nature of our analysis highlights a reciprocal relationship between parent–child communication about broad topics and identity-building STEM talk. This pattern is commensurate with the characteristics of “open family discussions” that define FCPT’s conversation orientation whereby “families holding this view value the exchange of ideas, and parents holding this belief see frequent communication with their children as the primary means by which to educate and to socialize them” (Koerner & Schrodt, 2014, p. 6). We found converging evidence supporting this interpretation in the significant relationship between our conversational variables and STEM identity given that conversation orientation is a strong predictor of psychosocial outcomes (ibid). However, we agree with Šimunović and Babarović (2020) that the “mechanisms through which parents may convey their STEM-related beliefs to their children are still unclear” (p. 701).

Exploring family conversational patterns broadens our ability to better model the culture-infused ways children in which are socialized into STEM, which we know occur very early in their development (Dabney et al., 2013; Maltese & Tai, 2010; Pattison et al., 2022; Paul et al., 2020; Tang et al., 2024). Motivated by their study of oral storytelling in Latine families, Haden et al. (2023) call for approaches to understanding children’s relationship to STEM that capitalize on shared “core values and lived experiences, among these the widespread preference for oral practices” (p. 4). By conceptualizing our structural model design through the framing of communication patterns, we avoided racialized group comparisons that often do more to perpetuate, rather than dismantle, structural racism associated with STEM contexts (McGee, 2016), while also encompassing the often-overlooked practices of culturally diverse families because of their presumed distal relation to STEM.

## 7. Conclusions

Our findings suggest that children whose caregivers fostered open communication in the home are more likely to develop STEM identities as a factor of the increased number of opportunities to engage in STEM talk. This adds significant complexity to our understanding of the social and familial factors that contribute to the development of STEM identity. By attending to parent–child conversations that do not fall neatly within a STEM-related categorization, we draw attention to the often under-recognized, identity supportive practices of caregivers. This research suggests that caregivers play a larger, more complex role in the development of children’s STEM identities than suggested in most of the STEM identity literature and calls for greater emphasis on family engagement in formal and informal STEM learning settings. Complemented by studies of programs that foster family conversations (e.g., Morris et al., 2023), our findings support a focus on efforts that invite parents and children to talk about their beliefs and experiences in and beyond STEM through open dialogue without placing undue expectations on parents’ involvement.

## Figures and Tables

**Table 1:** Family types characterized by patterns resulting from conversation and conformity orientations in Family Communication Patterns Theory (Koerner & Fitzpatrick, 2006).

Conversation Orientation	Conformity Orientation	Family Types	Communication Patterns
+	+	Consensual	Children tend to share parents’ beliefs. Parents seek their children’s agreement through open communication.
+	−	Pluralistic	Children and parents seek one another’s perspectives on a wide range of topics with little emphasis on agreement.
−	+	Protective	Conformity is expected through obedience with little consideration for divergent ideas.
−	−	Laissez-Faire	Family members show little concern for understanding or developing shared beliefs.

**Table 2:** Summary of respondent (*n* = 1134) population characteristics.

Respondent Characteristics	Response	Proportion
Institution Type	HBCU	0.56
	HSI	0.33
	Other	0.11
Gender	Female	0.54
	Male	0.41
	Non-Binary	0.02
	Prefer Not to Say	0.01
	Self-Described	0.02
Self-Reported Racial Identity	Black	0.44
	Hispanic	0.38
College Year	First Year	0.76
	Second Year	0.17
Academic Grade in Highest Secondary English Course	A	0.44
	B	0.36
	C or Below	0.08
	Cannot Recall	0.05
	No Grade or Missing	0.07
Academic Grade in Highest Secondary Mathematics Course	A	0.36
	B	0.33
	C or Below	0.20
	Cannot Recall	0.05
	No Grade or Missing	0.06
K—12 Institution Type	Public	0.80
	Private	0.10
	Home School	0.01
	Other	0.09

## Data Availability

For questions about data availability, please contact Phil Gerhard (psadler@cfa.harvard.edu).
